# Proliferating Astrocytes in Primary Culture Do Not Depend upon Mitochondrial Respiratory Complex I Activity or Oxidative Phosphorylation

**DOI:** 10.3390/cells12050683

**Published:** 2023-02-21

**Authors:** Ellen A. Silva, Ana P. Dalla Costa, Juliana S. Ruas, Edilene S. Siqueira-Santos, Annelise Francisco, Roger F. Castilho

**Affiliations:** Department of Pathology, School of Medical Sciences, State University of Campinas (UNICAMP), Campinas 13083-887, SP, Brazil

**Keywords:** astrocytes, bioenergetic, mitochondria, oligomycin, OXPHOS, piericidin A

## Abstract

Understanding the role of astrocytes in the development of the nervous system and neurodegenerative disorders implies a necessary knowledge of the oxidative metabolism of proliferating astrocytes. The electron flux through mitochondrial respiratory complexes and oxidative phosphorylation may impact the growth and viability of these astrocytes. Here, we aimed at assessing to which extent mitochondrial oxidative metabolism is required for astrocyte survival and proliferation. Primary astrocytes from the neonatal mouse cortex were cultured in a physiologically relevant medium with the addition of piericidin A or oligomycin at concentrations that fully inhibit complex I-linked respiration and ATP synthase, respectively. The presence of these mitochondrial inhibitors for up to 6 days in a culture medium elicited only minor effects on astrocyte growth. Moreover, neither the morphology nor the proportion of glial fibrillary acidic protein-positive astrocytes in culture was affected by piericidin A or oligomycin. Metabolic characterization of the astrocytes showed a relevant glycolytic metabolism under basal conditions, despite functional oxidative phosphorylation and large spare respiratory capacity. Our data suggest that astrocytes in primary culture can sustainably proliferate when their energy metabolism relies only on aerobic glycolysis since their growth and survival do not require electron flux through respiratory complex I or oxidative phosphorylation.

## 1. Introduction

Among the roles astrocytes play in the central nervous system, it is worth highlighting the metabolic support for neurons. To fulfill this metabolic role, astrocytes (i) degrade glucose to pyruvate, which is converted into lactate and supplied to neurons (astrocyte-neuron lactate shuttle); (ii) capture extracellular glutamate and convert it to glutamine (glutamate-glutamine cycle); and (iii) store glycogen (see [1,2,3,4] for review). The lactate shuttling between astrocytes and neurons allows the transference of the lactate produced during aerobic glycolysis within astrocytes to the neurons. In the neurons, lactate is used as a substrate for ATP generation through oxidative phosphorylation [1,5]. This mechanism may be meaningful for saving neuronal glucose, required in the pentose phosphate pathway [6], an important source of reducing equivalents in the form of NADPH for biosynthesis and redox homeostasis [7,8].

Many cell types use more aerobic glycolysis than mitochondrial oxidative phosphorylation even under typical oxygen tensions. Such a phenomenon was extensively studied in cancer cells, where enhanced glycolysis provides ATP and carbons to generate biomass during accelerated cell growth [9,10], even if the mitochondrial electron transport system and membrane integrity are preserved in most cancer cells [11,12,13]. Cancer cells may also switch from oxidative phosphorylation to glycolytic metabolism to decrease the production of reactive oxygen species [14]. Notably, cultured cancer cells were able to grow and survive even under the inhibition of oxidative phosphorylation by oligomycin [15]. Despite the prevalence of glycolysis, mitochondrial respiration remains important in proliferating cells, allowing the production of biosynthetic precursors and the generation of electron acceptors that enable aspartate synthesis, for example [9,16,17].

The primarily glycolytic metabolism of astrocytes has been previously demonstrated in non-proliferating astrocytes, which were able to survive without mitochondrial respiration [18]. The rate of glycolysis, determined by metabolic flux analysis, was significantly higher in primary astrocytes than in cultured neurons [6]. Conversely, neurons and astrocytes significantly used glucose as an energy substrate for ATP production via glycolysis and mitochondrial oxidative phosphorylation during periods of network activation in brain slices [19]. This apparent contradiction illustrates the gaps in the knowledge of metabolic peculiarities of the central nervous system. Assessing the mitochondrial bioenergetics in proliferating astrocytes has potential implications for a better understanding of the development and degenerative processes in the nervous system. Here, we demonstrated that proliferative mouse astrocytes in culture can sustainably proliferate when their energy metabolism relies only on aerobic glycolysis since their growth and survival do not require electron flux through respiratory complex I or oxidative phosphorylation.

## 2. Materials and Methods

### 2.1. Chemicals 

Carbonyl cyanide 3-chlorophenylhydrazone (CCCP; cat. C2759), dimethyl sulfoxide (DMSO; D8418), DL-dithiothreitol (DTT; D5545), dihydrate 4-(2-hydroxyethyl)piperazine-1-ethanesulfonic acid (HEPES; H3375), ethylenediaminetetraacetic acid disodium salt (EDTA; E5134), L-glutamine (G3126), oligomycin (O4876), rotenone (R8875), sodium dodecyl sulfate (SDS; L4509), sodium pyruvate (P4562), thiazolyl blue tetrazolium bromide (MTT; M5655), Triton™ X-100 (T9284), Trizma^®^ base (T1503), and trypan blue (T6146) were obtained from Merck (St Louis, MO, USA). Piericidin A (cat. 15379) was obtained from Cayman Chemical Company (Ann Arbor, MI, USA). Anti-glial fibrillary acidic protein (anti-GFAP) antibody (cat. PA3-16727) and AlexaFluor 488 goat anti-rabbit antibody (A-11008) were obtained from Thermo Fisher Scientific (Waltham, MA, USA). Antibiotics (penicillin and streptomycin); Dulbecco’s modified Eagle’s medium (DMEM) with 50 mg/L gentamicin sulfate, 25 mg/L amphotericin B, 15 mg/L phenol red, and with or without 5.5 mM glucose; 1 mM pyruvate; and 4 mM glutamine came from Vitrocell (Campinas, SP, Brazil). Fetal bovine serum (FBS), phosphate-buffered saline (PBS), and trypsin/EDTA solution (0.25%) were also purchased from Vitrocell. Stock solutions of CCCP, oligomycin, and piericidin A were prepared by dissolving the respective chemicals in DMSO. Rotenone and antimycin solutions were prepared by dissolving the respective chemicals in ethanol. HEPES buffer was prepared in ultrapure water, and the pH was adjusted to 7.2 with NaOH. Glutamine and pyruvate were prepared in water, and the pH was adjusted to 7.4 with NaOH. 

### 2.2. Animals 

Postnatal day 1–2 mouse pups were from a breeding colony of C57BL/6JUnib mice obtained from the Campinas University Multidisciplinary Center for Biological Research in Laboratory Animal Science (CEMIB/UNICAMP, Campinas, Brazil). Breeding mice were kept in monogamous pairs under standard laboratory conditions (22–24 °C and 12/12-h light/dark cycle), with free access to filtered water and a standard diet (Nuvilab CR1, Nuvital, Colombo, PR, Brazil). The use of mice and the experimental protocols received approval from the local Ethics Committee on Animal Research (CEUA) of UNICAMP, under approval number 5642-1/2020. The animal procedures complied with the guidelines of the National Council for Control in Animal Experiments (CONCEA), the Guide for the Care and Use of Laboratory Animals (National Institutes of Health), and the Animal Research: Reporting of In Vivo Experiments (ARRIVE) guidelines.

### 2.3. Primary Astrocyte Culture 

Primary astrocytes were obtained from mouse pups at postnatal day 1 or 2 as described elsewhere [20,21]. The cells obtained after homogenization of mouse pup cortices and successive centrifugations of the cell suspension were plated in a 75 cm^2^ culture flask with Cell+ Surface filter (Sarstedt, Nümbrecht, Germany) with DMEM containing 5.5 mM glucose, 4 mM glutamine, 1 mM pyruvate, 100 IU/mL penicillin, 100 µg/mL streptomycin, and 10% FBS and incubated at 37 °C with 5% CO_2_. After reaching 80 to 100% confluence, the culture flask was shaken at 240 rpm for 5–6 h in a culture medium containing 20 mM HEPES-Na^+^. Then, the astrocytes that remained adhered to the flask were trypsinized, plated in new T75 culture flasks, and maintained at 37 °C in the CO_2_ incubator. Astrocytes at passages 2 and 3 were used for downstream experiments.

### 2.4. Culture Medium Optimization 

Astrocytes were plated at 30,000 cells/cm^2^ density in a 24-well plate (1.93 cm^2^/well) and incubated for up to 7 days in 2 mL of DMEM supplemented with 100 IU/mL penicillin, 100 µg/mL streptomycin, and 10% FBS, containing different concentrations of metabolic substrates: 25 mM glucose, 4 mM glutamine, and 1 mM pyruvate (Medium 1); 5.5 mM glucose, 4 mM glutamine, and 1 mM pyruvate (Medium 2); or 5.5 mM glucose, 0.8 mM glutamine, and 0.3 mM pyruvate (Medium 3). The above-mentioned metabolic substrate concentrations refer to the final concentrations in the medium. The cells were plated in duplicates and analyzed concerning cell number and viability after one (D1), three (D3), five (D5), and seven (D7) days in culture. 

### 2.5. Oxygen Consumption Rate (OCR) Assessment 

In the first set of experiments, the OCR in intact astrocytes was determined using a high-resolution oxygraph (Oroboros Oxygraph-2k, Innsbruck, Austria). For this, the astrocytes were trypsinized and suspended in Medium 3 containing 20 mM HEPES-Na^+^. Cells (7.5 × 10^5^) were added to the 2 mL oxygraph chamber containing Medium 3 plus 20 mM HEPES-Na^+^. Additions of mitochondrial inhibitors oligomycin or piericidin A were performed every 2 to 3 min until reaching the final concentration of 2 µg/mL oligomycin and 4 µM piericidin A. In some experiments, 2 µM rotenone and 1 µM antimycin were also added.

To assess the chronic inhibitory effect of the mitochondrial inhibitors oligomycin and piericidin A on the adherent cell culture, 60,000 cells/well were plated in the Seahorse XFe24 24-well analyzer (Agilent Technologies, Santa Clara, CA, USA) plate with 500 µL of Medium 3 at D0. A high cell density was employed in these experiments due to equipment’s requirements. DMSO (0.025%, *v/v*; solvent—control condition), 2 µM piericidin A, or 1 µg/mL oligomycin were added to the respective treatment wells from the first day (D1) of culture and incubated for 48 h. One hour before the experiment, HEPES-Na^+^ 20 mM was added to each well without changing the culture medium, and the plate was incubated at 37 °C in the absence of CO_2_. Injections containing 75 µL of Medium 3 or mitochondrial inhibitors diluted in Medium 3 were performed to induce the desired experimental conditions for OCR evaluation. The effective mitochondrial OCR inhibition was assured by additions of 1 µM rotenone plus 1 µM antimycin. The OCR values of each well were normalized by the total DNA content determined by crystal violet staining. The presence of sodium bicarbonate in the medium precluded performing assessment of extracellular acidification rate (ECAR) in this experimental set.

### 2.6. Chronic Treatments of Cell Culture with Mitochondrial Inhibitors 

Oligomycin (1 µg/mL) and piericidin A (2 µM) were present in the culture medium from day one (D1). DMSO (0.025%, *v/v*; solvent) was used as a negative control. For this, 30,000 cells/cm^2^ were plated in a 24-well plate in 2 mL of Medium 3 and incubated for up to six days with the different compounds. The astrocytes were plated in duplicates and analyzed concerning cell number and viability at D1, D4, and D7. 

### 2.7. Cell Counting 

To count the viable cells, the culture medium was removed, and the adhered astrocytes were washed with phosphate-buffered saline (PBS, pH 7.4), trypsinized, and centrifuged. The cell pellet was suspended in Medium 3 and diluted 1:10 in 0.4% trypan blue and then viable cells were counted in a Neubauer chamber.

### 2.8. ATP Content 

Cell viability was evaluated by measuring ATP content using the CellTiter-Glo assay (cat. G7571, Promega, Madison, WI, USA) as described previously [20,22]. Briefly, the culture medium was removed, and the adhered astrocytes were washed with PBS, trypsinized, and centrifuged at 400× *g* for 5 min. Then, astrocytes were lysed by adding 1 mL of a lysing solution (25 mM Tris buffer pH 7.8, 2 mM DL-dithiothreitol, 2 mM EDTA, 10% glycerol, and 1% Triton X-100). The ATP content of the cell lysate was measured by luminescence following the manufacturer’s instructions.

### 2.9. MTT Assay 

Cell viability was assessed from the reduction of thiazolyl blue tetrazolium bromide (MTT) to blue-colored formazan crystals by metabolically active cells in the culture, as described previously [20,23]. Briefly, the culture medium was removed from the wells, and the adhered astrocytes were washed with PBS. Then, 400 µL of 1 mg/mL MTT solution in phenol red-free DMEM was added to the adhered astrocytes and incubated for 90 min at 37 °C with 5% CO_2_. After incubation, 400 µL of SDS solution (10% SDS in 0.01 M HCl) was added to each well and incubated overnight. The amount of formazan generated was determined spectrophotometrically by differential absorbance at 570–650 nm.

### 2.10. Lactate Dehydrogenase (LDH) Release 

To quantify the LDH presence in the culture medium after incubation under the different experimental conditions, 30,000 cells/cm^2^ were plated in a 24-well plate in 2 mL of phenol red-free Medium 3 supplemented with DMSO (0.025%, *v/v*; solvent—control condition), 2 µM piericidin A, or 1 µg/mL oligomycin since D1. The culture media were changed at D1 and D4, and the samples of astrocytes culture medium were collected 72 h after the media changes (i.e., D4 and D7). LDH activity was estimated using a commercial enzymatic-colorimetric kit (LDH K014-2, Quibasa Química Básica Ltda—Bioclin, Belo Horizonte, Brazil). The total LDH content was determined after cell lysis with 1% Triton X-100.

### 2.11. Glucose Consumption and Lactate Release 

Glucose and lactate concentrations in the culture medium were measured using commercial enzymatic-colorimetric kits (K082-2 and K084-2, Quibasa Química Básica Ltda—Bioclin, Belo Horizonte, Brazil). Astrocytes were seeded at an initial density of 60,000 cells/cm^2^ in a 24-well plate in 2 mL of phenol red-free Medium 3. DMSO (0.025%, *v/v*; solvent), 2 µM piericidin A, or 1 µg/mL oligomycin were added at D1. Cells were maintained in culture for an additional 24 h (D2), when samples of astrocyte culture medium were collected and analyzed. Viable cells were counted with trypan blue at D1 and D2; by means of these measurements, we estimated the mean cell number during the incubation as 74,000 cells/cm^2^. This value was used for calculation of ATP regeneration by glycolysis as a function of lactate release. 

### 2.12. Immunocytochemistry 

Astrocytes were plated at 26,000 cells/cm^2^ in an 8-well Permanox plastic slide (0.8 cm^2^/well, Nunc Lab-Tek Chamber Slide System, cat. 177445, Thermo Fisher Scientific, Waltham, MA, USA) in 0.4 mL of Medium 1, 2, or 3 since D0, or incubated in Medium 3 with 1 µg/mL oligomycin, 2 µM piericidin A, or DMSO (0.025%, *v/v*; solvent—control condition) from D1. The medium was changed at D3 maintaining the same experimental conditions. After 5 days, astrocytes were washed with PBS and fixed with a buffer containing 4% paraformaldehyde and 4% sucrose for 15 min at room temperature. Next, the astrocytes were incubated in PBS containing 0.8% Triton X-100, and 3% BSA for 1 h at room temperature. Immunolabeling was performed with the rabbit anti-GFAP primary antibody (1:500) for 16 h at 4 °C, followed by AlexaFluor 488 goat anti-rabbit antibody (1 μg/mL) for 2 h at room temperature, and 1 μg/mL DAPI for 10 min. Slides were mounted with H-100 Vectashield (Vector Laboratories Inc., Newark, CA, USA) and analyzed using a fluorescence microscope (NIKON Eclipse 80i, Nikon Corporation, Tokyo, Japan). 

### 2.13. Statistical Analysis 

Data are presented as representative traces, individual means of experimental duplicates (empty circles), and bars representing the mean + standard deviation. Sample sizes are described in the figure legends. The normality distribution was verified using the Shapiro-Wilk test. Statistical analyzes employed Prism 7 software (GraphPad Software, Boston, MA, USA) using the one- or two-way ANOVA tests, followed by Bonferroni’s post hoc, or Kruskal–Wallis with Dunn’s multiple comparisons test where indicated. A minimum significance level of *p* ˂ 0.05 was considered. 

## 3. Results

### 3.1. High Concentrations of Metabolic Substrates Are Not Required to Maintain Astrocyte Growth and Viability

Primary astrocytes are usually cultured in media with metabolic substrates at concentrations higher than those present in the cerebrospinal fluid (CSF) despite the scarcity of standardization on the optimal composition of the culture media to evaluate the bioenergetic metabolism of astrocytes [24]. Here, primary astrocytes were first cultured in DMEM with different metabolic substrate concentrations to evaluate if changes in medium composition would impair astrocyte growth and survival.

**Figure 1:**
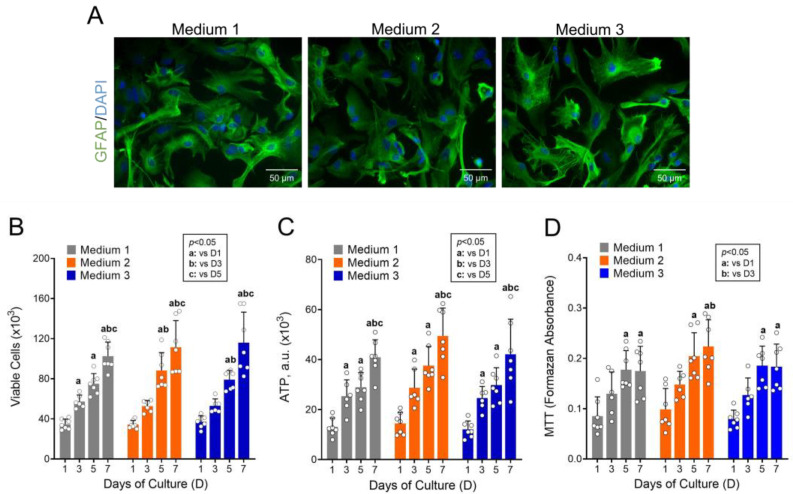
Proliferation and viability of astrocytes cultured in media containing different concentrations of energy substrates. Astrocytes were cultured in DMEM containing various concentrations of energy substrates, such as 25 mM glucose, 4 mM glutamine, and 1 mM pyruvate (Medium 1); 5.5 mM glucose, 4 mM glutamine, and 1 mM pyruvate (Medium 2); 5.5 mM glucose, 0.8 mM glutamine, and 0.3 mM pyruvate (Medium 3). Cells were analyzed after one (D1), three (D3), five (D5), and seven (D7) days in culture. (**A**) Microphotographs of astrocytes at D5 when cultured in Media 1, 2, and 3. (**B**) Cell viability by trypan blue. (**C**) ATP content in cultured cells. a.u.: Arbitrary units. (**D**) Cell viability by MTT. Statistically significant by two-way ANOVA, post-hoc Bonferroni (*p* < 0.05); “a” compared to D1, “b” compared to D3, and “c” compared to D5 in the same medium. N = 6–7.

Figure 1A shows microphotographs of astrocytes cultured during 5 days in Medium 1, 2, and 3 and stained with anti-GFAP (green) and DAPI (blue), labeling astrocytic cytoskeleton and nucleus, respectively. It is worth noting that nearly all astrocytes in the culture were strongly GFAP-positive, suggesting a reactive phenotype. The culture in these different media did not elicit any noticeable morphological change in astrocytes. The growth and survival of astrocytes did not differ among Media 1, 2, and 3 when viability underwent evaluation by trypan blue (Figure 1B), total ATP content in cells (Figure 1C), and MTT assay (Figure 1D). 

From these data, the growth and viability of cultured astrocytes were also compared across time (D1, D3, D5, and D7) within the same experimental condition. Cell proliferation increased by 2.0 to 3.5 times from D1 to D7, being significant from D3 or D5 when compared with D1 in the evaluations by trypan blue exclusion dye and ATP content and from D5 in the evaluation by MTT reduction. Cell viability by trypan blue was above 85% in all evaluated conditions and did not differ among groups, regardless of the medium and days in culture. Since the different media did not change the growth and viability of astrocytes, Medium 2 was chosen for the maintenance of astrocytes in flasks during cell culture to avoid depleting metabolic substrates and cellular stress over long periods under a low volume of medium per culture area. Medium 3 was used to carry out the experiments since its concentrations of glucose, glutamine, and pyruvate were closer to the physiological concentrations of these substrates in the cerebrospinal fluid.

### 3.2. The Effect of the Mitochondrial Inhibitors Oligomycin and Piericidin A on the Rate of Astrocytes’ Oxygen Consumption (OCR)

The concentrations of the mitochondrial inhibitors oligomycin and piericidin A that fully inhibit, respectively, ATP synthase and complex I-linked respiration were estimated by the effects of titration of these compounds in the OCR of astrocytes. 

**Figure 2:**
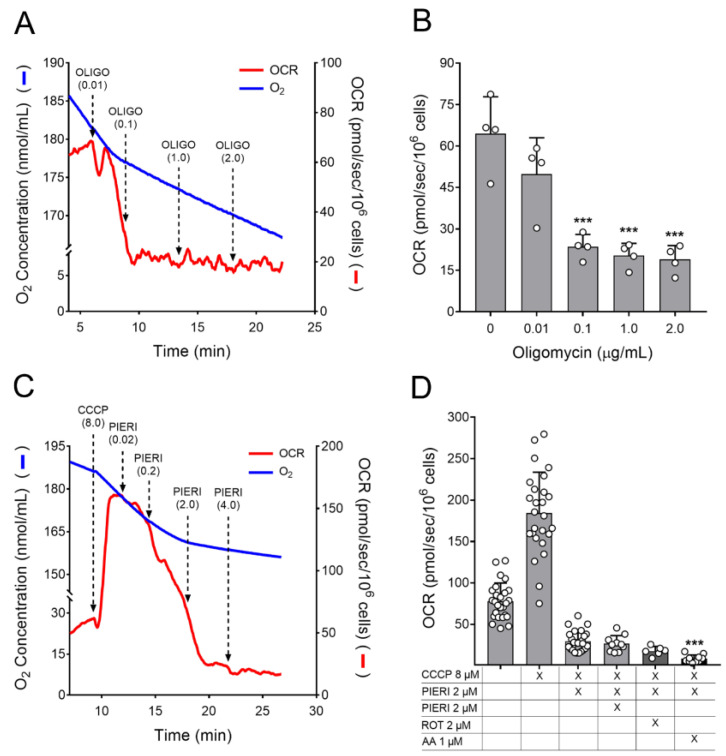
Effect of oligomycin and piericidin A on the oxygen consumption rate (OCR) of astrocytes in suspension. Astrocytes (7.5 × 10^5^/mL) were resuspended in Medium 3 containing 20 mM HEPES-Na^+^, and OCR was monitored in a high-resolution oxygraph. (**A**) Representative trace of OCR in suspended astrocytes. As indicated by the arrows, oligomycin (OLIGO) additions were made until reaching the final concentration of 2.0 µg/mL. The oligomycin concentrations in µg/mL achieved after each addition are shown in parentheses. (**B**) OCR values obtained after sequential additions of oligomycin. *** Statistically different from basal in the absence of oligomycin, by one-way ANOVA, post-hoc Bonferroni, *p* < 0.001. (**C**) Representative trace of OCR in suspended astrocytes. As indicated by the arrows, additions of piericidin A were made until reaching a final concentration of 4.0 µM. The piericidin A concentrations in µM achieved after each addition are shown in parentheses. (**D**) OCR values obtained under basal condition and after additions of 8 µM CCCP, piericidin A (PIERI; 2 and 4 µM) plus rotenone (ROT; 2 µM) or antimycin (AA; 1 µM) as indicated. Statistically different from OCR in the presence of 8 µM CCCP plus 2 µM piericidin A, by Kruskal-Wallis followed by Dunn’s multiple comparisons test, *** *p* < 0.001.

To evaluate the inhibition of ATP synthase by oligomycin (Figure 2A,B), sequential additions of this inhibitor were performed until reaching the final concentration of 2.0 µg/mL. The representative trace (Figure 2A) illustrated that a progressive inhibition of OCR occurred from adding 0.01 µg/mL of oligomycin, and a maximum inhibition was achieved in the presence of 0.1 µg/mL oligomycin. The OCR values of astrocytes obtained before and after adding oligomycin at final concentrations of 0.01, 0.1, 1.0, and 2.0 µg/mL oligomycin are represented in bars in Figure 2B. Statistical analysis of the OCR values obtained after additions of oligomycin, compared to the value obtained in the absence of the inhibitor, showed a significant inhibitory effect from 0.1 µg/mL oligomycin. Even with this result, 1 µg/mL oligomycin was chosen to keep up the experiments with astrocytes in culture since the use of this oligomycin concentration is common in the literature and causes a minor nonspecific effect on mitochondrial and cellular function [15,25,26,27].

To assess the inhibition of respiratory complex I by piericidin A, maximal OCR should be initially reached, avoiding underestimation of the inhibition threshold. The maximal OCR was determined by titration of the protonophore CCCP (Supplementary Figure S1), where the maximal OCR stimulus was observed with 10 µM CCCP. However, to avoid the occurrence of OCR inhibition due to CCCP overload and long incubation times [26], a suboptimal concentration of CCCP (i.e., 8 µM) was used to stimulate the OCR in the experiments of the inhibition of complex I by piericidin A.

According to the representative trace in Figure 2C, after inducing the maximal OCR with CCCP, sequential additions of piericidin A were performed until reaching a final concentration of 4 µM piericidin A. A progressive inhibition of OCR was induced by adding 0.02 µM piericidin A, and a maximum inhibition was achieved a few minutes after the addition of 2.0 µM piericidin A. To ensure that 2 µM piericidin A was sufficient to obtain maximum inhibition of complex I; in independent experiments, a further addition of 2 µM piericidin A, 2 µM rotenone (complex inhibitor respiratory I), or 1 µM antimycin (respiratory complex III inhibitor) was made. Figure 2D shows the OCR values obtained after the sequential additions of these mitochondrial inhibitors. In this set of experiments, the presence of 4 µM piericidin A did not result in additional inhibition of OCR. A non-significant additional inhibition can be observed after the addition of 2 µM rotenone. However, this trend may be partially due to a long time in the run. The presence of 1 µM antimycin caused a further inhibition of OCR due to an arrest of the electron transport system caused by respiratory complex III inhibition. In light of these results, the concentration of 2 µM piericidin A was chosen for the experiments of astrocytes in culture. The use of this piericidin A concentration does not cause the unspecific effects that could be observed under higher concentrations of piericidin A, such as the inhibition of respiratory complex III [28]. 

**Figure 3:**
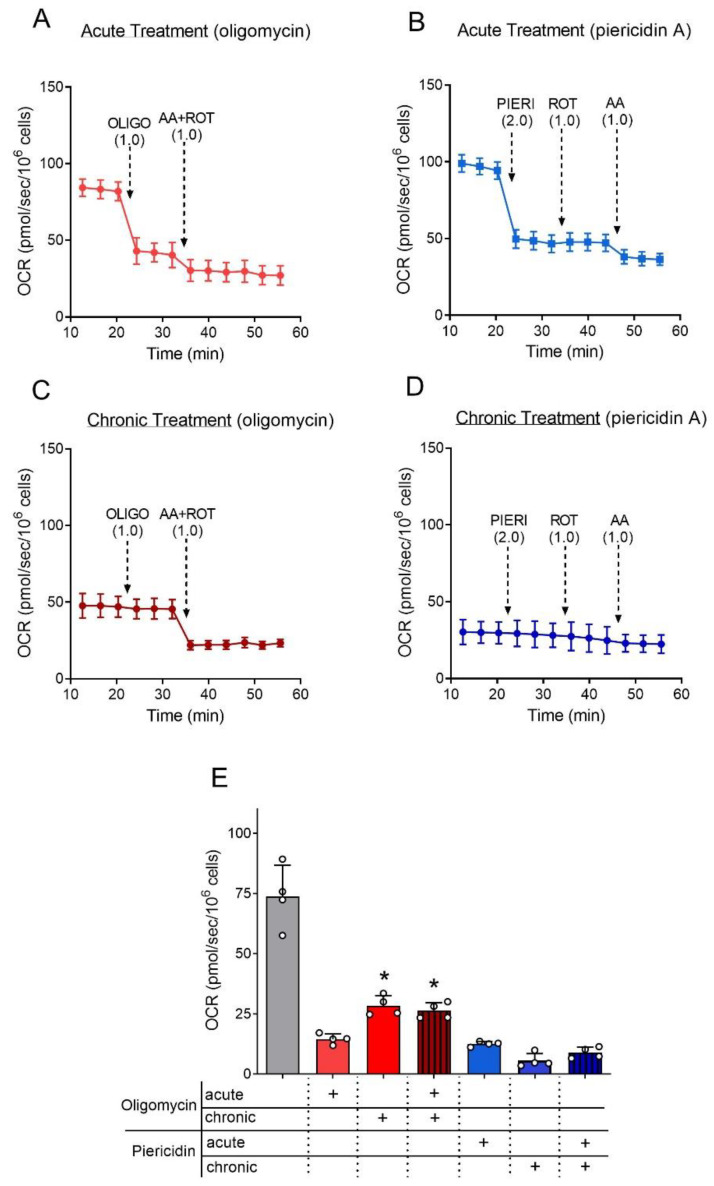
Effect of oligomycin and piericidin A on the oxygen consumption rate (OCR) of adhered astrocytes. Astrocytes (60,000 cells/well) were plated in a 24-well plate specific for use in the Seahorse XFe24 analyzer and cultured in Medium 3 with DMSO (0.025%, *v/v*; solvent) or the mitochondrial inhibitors oligomycin (1 µg/mL) and piericidin A (2 µM) present from D1. Cells were evaluated after 48 h (D3) when oligomycin or piericidin A were also added during the runs. (**A**) Representative trace of OCR of adhered astrocytes treated with DMSO and acutely treated with oligomycin. Where indicated, a bolus addition of 1.0 µg/mL oligomycin (OLIGO) was performed, followed by 1 µM antimycin plus 1 µM rotenone (AA + ROT). (**B**) Representative trace of OCR of adhered astrocytes treated with DMSO and acutely treated with piericidin A. Where indicated, a bolus addition of 2.0 µM piericidin A (PIERI) was performed, followed by ROT and AA. (**C**) Representative trace of OCR by adhered astrocytes chronically treated with oligomycin. Where indicated, additions of OLIGO, ROT, and AA were made. (**D**) Representative trace of OCR by adhered astrocytes chronically treated with piericidin A. Where indicated, additions of PIERI, ROT and AA were made. (**E**) Antimycin-sensitive OCR values from adhered astrocytes in basal condition, chronically and/or acutely treated with piericidin A and oligomycin. * Statistically significant by two-way ANOVA, post-hoc Bonferroni, compared to Oligomycin (acute). *p* < 0.05. N = 4.

Assessing the acute and chronic effects of oligomycin and piericidin A on the OCR of adhered astrocytes represents an important step in determining whether the presence of these compounds for a long time in a culture medium would reduce the effectiveness of inhibiting mitochondrial complexes. In Figure 3, OCR was estimated in adherent astrocytes using the Seahorse analyzer. The acute effects of oligomycin and piericidin A were assessed by adding these mitochondrial inhibitors to cultured astrocytes during the runs (Figure 3A,B). Both inhibitors elicited an extensive inhibition of mitochondrial respiration. In experiments with long incubation times (4 to 7 days), the culture medium with mitochondrial inhibitors was changed every 48 h. Thus, to assess the chronic effects of mitochondrial inhibitors on OCR, astrocytes were cultured for 48 h in the presence of 1 µg/mL oligomycin, 2 µM piericidin A or DMSO (0.025%, *v/v*). The representative trace in Figure 3C shows that the injection of 1 µg/mL oligomycin did not cause further inhibition of OCR in cells chronically treated with oligomycin. An additional inhibition of OCR was observed after the injection of 1 µM antimycin plus 1 µM rotenone due to the arrest of the electron transport system. A similar profile was observed in cells previously treated with piericidin A (Figure 3D); the acute injection of 2 µM piericidin A did not elicit any additional inhibition of OCR. In line with expectations, the injection of 1 µM rotenone had no additional effect on OCR while the addition of 1 µM antimycin further inhibited OCR. 

The OCR values obtained after chronic and/or acute treatment with oligomycin and piericidin A in adherent astrocytes are presented in Figure 3E. The chronic treatment with oligomycin caused a significantly lower inhibition of OCR when compared with the acute treatment. However, adding oligomycin in astrocytes chronically treated with this compound did not further inhibit OCR. In astrocytes treated with piericidin A, no significant difference in OCR was observed due to chronic or acute treatment. This experiment showed that the OCR inhibition by oligomycin and piericidin A were effective during incubation. However, the oligomycin-insensitive fraction increased in chronic exposure to this inhibitor.

### 3.3. Astrocytes Can Grow and Survive despite the Inhibition of ATP Synthase and Respiratory Complex I

**Figure 4:**
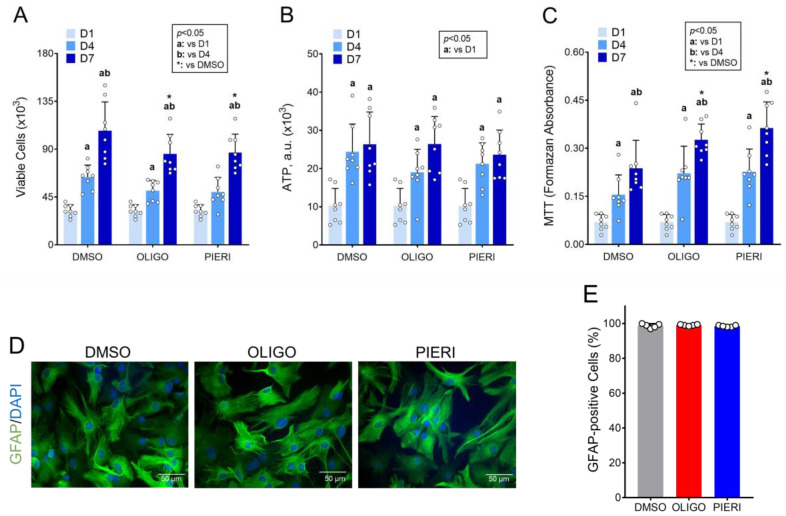
Proliferation and viability of astrocytes cultured in the presence of mitochondrial inhibitors. Astrocytes were cultured in Medium 3, containing DMSO (0.025%, *v/v*; solvent), oligomycin (OLIGO; 1 µg/mL), or piericidin A (PIERI; 2 µM) from D1. Cells were analyzed after one (D1), four (D4), and seven (D7) days in culture. (**A**) Cell viability by trypan blue. (**B**) ATP content in cultured cells. a.u.: Arbitrary units. (**C**) Cell viability by MTT. (**D**) Microphotographs of astrocytes cultured for four days in the presence of DMSO, OLIGO, or PIERI. (**E**) Percentage (%) of glial fibrillary acidic protein (GFAP)-positive astrocytes within cells cultured in the presence of mitochondrial inhibitors. Cells were analyzed after four days in culture. Statistically significant by two-way ANOVA, post hoc Bonferroni (*p* < 0.05); “a” compared to D1, “b” compared to D4, * compared to control (DMSO) at the same time point. N = 5–8.

The growth and viability of proliferating astrocytes cultured in Medium 3 supplemented with oligomycin or piericidin A were assessed by counting viable cells stained with trypan blue (Figure 4A), total ATP content in cultured cells (Figure 4B), and the MTT assay (Figure 4C). When cell proliferation by trypan blue was compared across time within the same treatment, the cell number increased 3.3 times from D1 to D7 in the DMSO condition while a 2.6-fold increase was observed when astrocytes were cultured with oligomycin or piericidin A. When measuring ATP content, an increase of approximately 2.5 times was observed from D1 to D7 independent of treatment. In the MTT assay, cell proliferation increased by 3.4, 4.7, and 5.2 from D1 to D7 in the DMSO, oligomycin, and piericidin A treatment, respectively. In all analyses, astrocyte growth was significant from D4, except for astrocytes incubated in the presence of piericidin A and analyzed by trypan blue, whose growth was significant just from D7. These data show that astrocytes can proliferate regardless of the presence of mitochondrial inhibitors.

When oligomycin and piericidin A treatments were compared with DMSO controls, cell growth evaluated by trypan blue significantly decreased by 20% after 7 days in the presence of mitochondrial inhibitors (Figure 4A). No difference among treatments was observed when ATP content was used to estimate cell proliferation (Figure 4B). Conversely, cell proliferation measured by MTT reduction was 37% and 53% higher in astrocytes cultured for 7 days in the presence of oligomycin and piericidin A, respectively (Figure 4C). The increase in the reduction of MTT in the presence of mitochondrial inhibitors may be explained in terms of the more reduced state of NAD in this condition and does not represent an actual increase in cell proliferation, as elicited when considering viable cell counting and ATP content assay. 

The GFAP labeling of astrocytes cultured in the presence of oligomycin and piericidin A (Figure 4D) showed that these treatments did not affect astrocyte morphology. Moreover, this labeling ruled out the possibility that the measured cell growth was due to the proliferation of non-astrocytic cells. More than 95% of the cells were positive for GFAP both in control conditions and in the two treatments (Figure 4E). 

**Figure 5:**
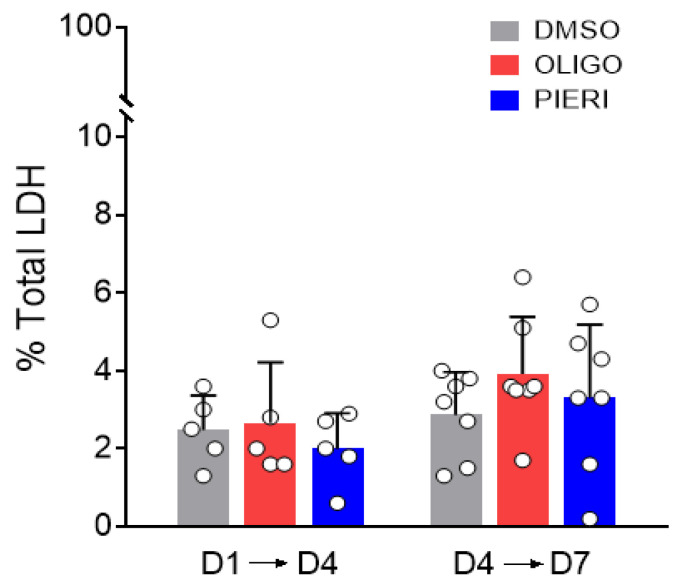
Lactate dehydrogenase (LDH) concentration in the medium after culturing astrocytes with mitochondrial inhibitors. Astrocytes were cultured in Medium 3, containing DMSO (0.025%, *v/v*; solvent), oligomycin (OLIGO; 1 µg/mL), or piericidin A (PIERI; 2 µM) from day one (D1). The culture media were replaced at days one (D1) and four (D4). Therefore, media for LDH quantification were collected 72 h after medium replacement, thus at D4 (D1 → D4) and/or at D7 (D4 → D7). N = 5–7.

The 20% lower growth in cells cultured with oligomycin and piericidin A observed in evaluations with trypan blue might be explained by increased cell death. To address this issue, the activity of LDH was measured in the astrocyte culture medium at D4 and D7. LDH is an enzyme inside the cell released when cell death occurs, and the integrity of the cell membrane is lost [29]. Figure 5 shows no statistical difference in LDH activity in the medium when comparing oligomycin and piericidin A conditions to the control condition. Thus, the presence of mitochondrial inhibitors in the culture medium did not induce cell death other than the cell death resulting from the plating process and normal cell aging. Taken together, the results indicate that the presence of oligomycin and piericidin A did not affect cell survival and had minor effects on astrocyte growth.

### 3.4. Proliferating Astrocytes Consume Glucose and Release Lactate to Similar Extents

Despite the inhibition of oxidative phosphorylation in astrocytes cultured with oligomycin or piericidin A, changes in ATP content were not observed under these conditions (Figure 4B). Under these circumstances, increased glycolysis might compensate for the shortage of ATP from mitochondria. To test this hypothesis, glucose and lactate in the culture medium were quantified after 24 h incubation with DMSO, oligomycin, or piericidin A. 

**Figure 6:**
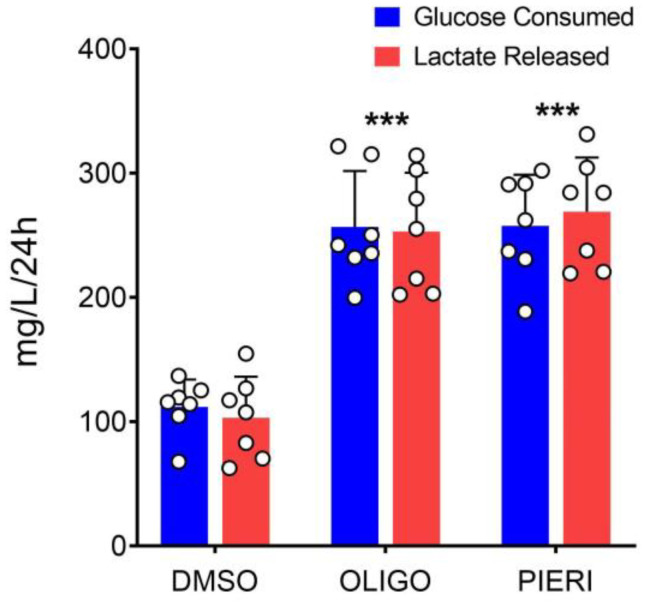
Glucose consumption and lactate released in astrocytes cultured in the presence of mitochondrial inhibitors. Astrocytes were cultured in Medium 3, containing DMSO (0.025%, *v/v*; solvent), oligomycin (OLIGO; 1 µg/mL), or piericidin A (PIERI; 2 µM) from day one (D1). Culture medium samples for glucose and lactate quantification were collected after 24 h of the addition of mitochondrial inhibitors to cultured astrocytes. For more details, see Section 2. *** Statistically significant by one-way ANOVA and the Bonferroni compared to the respective DMSO condition (*p* < 0.001). N = 7.

Figure 6 shows a significant increase in glucose consumption and lactate release in the presence of respiratory chain inhibitors. In the presence of oligomycin, glucose consumption increased by 129%, and the lactate release increased by 145%. When piericidin A was in the medium, glucose consumption increased by 130%, and the lactate release increased by 160%. Notably, data in Figure 6 also indicates that proliferating astrocytes consume glucose and release lactate to similar extents even in the control condition. 

## 4. Discussion

Proliferating astrocytes are remarkably present and active during the development of the nervous system, but they are also relevant to the mature nervous system, by taking part in the reactive gliosis induced by brain injury [30,31], the formation of the glial scar after spinal cord injury [32], and the physiopathology of neurodegenerative diseases [33,34]. Metabolic factors may be key determiners of the capacity of astrocytes to proliferate. In this sense, understanding the role of astrocytes in the development of the nervous system and neurodegenerative disorders implies a necessary knowledge of the oxidative metabolism of proliferating astrocytes. It is worth highlighting that our results are more valid in a context of reactive astrocytes since pure astrocyte cultures present typically reactive phenotype. Accordingly, the astrocytes obtained here presented a GFAP positive profile, regardless culture medium or treatment (Figure 1A and Figure 4D).

When performing metabolic evaluations in cell cultures, the concentration of metabolic substrates in the culture medium can significantly interfere with the results. Many studies have cultured primary human and rodent astrocytes in media with high concentrations of glucose (e.g., [20,21,35]). However, primary astrocytes cultured under high concentrations of glucose presented cell cycle arrest and inhibited proliferation, migration, and metabolic changes [24]. This study draws attention to the need to review the concentration of metabolic substrates in the culture medium to avoid excessive amounts of substrates, especially glucose. In this sense, we evaluated the growth and viability of primary astrocytes cultured with a medium containing different concentrations of glucose, pyruvate, and glutamine (Figure 1). Here, glucose concentration in the culture medium did not interfere with the growth and survival of astrocytes. This discrepancy between our results and the findings of Lee et al. [24] may be due to the higher plating density used in the present study (30,000 cells/cm^2^ vs. ~12,500 cells/cm^2^, respectively). Plating density can change the morphology and survival of astrocytic and neuronal cultures [36,37] and can also be related to the previously reported greater susceptibility of astrocytes to high glucose. Moreover, our results support that primary astrocytes can be cultured with lower concentrations of glucose, closer to the physiological ones.

Because the different media tested did not change the growth and viability of astrocytes, we chose to use the medium with a lower concentration of metabolic substrates to reduce other biases. The experiments were carried out in DMEM with glucose present in concentrations above those found in the cerebrospinal fluid, but similar to the glucose concentration in the plasma. Glutamine and pyruvate were also present in concentrations resembling the physiological ones [38,39,40]. Notably, a small substrate surplus was used to avoid substrate shortage during long incubation times. Extracellular pyruvate has been demonstrated to be an important mitochondrial energy substrate for cultured astrocytes [41]. It is worth noting that pyruvate supplementation also allows cells to live in the absence of mitochondrial respiration because, in this case, oxidation of NADH by lactate dehydrogenase can occur in the absence of a mitochondrial respiratory chain. The availability of NAD^+^ as an electron acceptor enables aspartate synthesis through aspartate aminotransferase and rescues the proliferation of cultured cells with dysfunctions or inhibition of the electron transport system [16,17]. Additionally, when the electron transport system is inhibited, reversed carbon flux through the mitochondrial dehydrogenases of Krebs cycle can provide oxidized redox equivalents to support glutamate dehydrogenase activity and cell viability [42].

Mitochondrial inhibitors were also titrated to find the minimal concentration able to inhibit mitochondrial respiration (Figure 2). Oligomycin inhibits ATP synthesis by binding to the subunit-c chain in contact with the proton half-channels formed by subunit-a of ATP synthase [43]. Piericidin A inhibits the mitochondrial NADH:ubiquinone oxidoreductase (complex I) by binding at or close to the ubiquinone binding site of the enzyme [44]. Based on this titration, as little as 0.1 µg/mL oligomycin would significantly inhibit mitochondrial respiration. However, we used 10 times more oligomycin in our experiments since this oligomycin concentration elicits minor nonspecific effects and it is common in the literature [15,25,26,27]. On the other hand, the piericidin A concentration used was the minimum required to reach a maximal inhibitory effect. Even if a slightly better inhibitory effect could be reached with 4 µM piericidin A, concentrations of this compound above 2 µM can inhibit the ubiquinol-citochrome c reductase activity of complex III [28]. The inhibition of mitochondrial respiration promoted by oligomycin and piericidin A was effective and lasted during all time under culture (Figure 3). This experimental set also shows that astrocytes do not become resistant to inhibition, or the compounds degraded during the cultivation. It is worth noting that rotenone, the most known complex I inhibitor, was not used in the present study since rotenone affects cytoskeleton stability and organization [45], thereby affecting cell differentiation and growth by a diverse mechanism. Of note, the respiratory rates measured at basal condition were similar between suspended and attached astrocytes (Figure 2B,D vs. Figure 3A,B), in line with previous observations on human fibroblasts [46].

Once the concentrations of substrates and inhibitors were optimized, the growth and viability of primary astrocytes cultured were evaluated by trypan blue dye exclusion staining and MTT reduction assay. Moreover, ATP quantification was used to indirectly estimate the number of the cells in a culture (Figure 4). Despite a 20% lower proliferation detected with cell counting by trypan blue dye, our data show that the presence of mitochondrial inhibitors does not affect astrocyte proliferation. Slight differences in the output obtained with the different techniques of cell viability assessment can occur due to the particularities of these approaches. In the presence of piericidin A, for example, the NAD can become highly reduced, causing a stronger reduction of MTT to formazan by dehydrogenases, thereby producing an increase in this measurement that does not reflect an increase in cell proliferation. Moreover, the presence of oligomycin or piericidin A did not elicit noticeable morphological changes or increased cell death in cultured astrocytes (Figure 5). 

These results raise a question concerning how astrocytes survive in the presence of piericidin A and oligomycin. In the presence of these inhibitors, the highly reduced state of components of the electron transport system causes a rise in the production of superoxide at these sites [47,48], which would be a challenge to cell survival. In our experiments, astrocytes chronically treated with oligomycin showed larger non-phosphorylating respiration (H^+^ leak). The increased proton conductance of the mitochondrial membrane may help astrocytes to decrease the mitochondrial superoxide production [47] and oxidate NADH. In the presence of piericidin A, this effect was not observed, and possibly other mechanisms have been established to circumvent the increased superoxide production due to complex I inhibition. Moreover, the conversion of pyruvate to lactate could supply oxidized NAD to glycolysis, thereby sustaining the cell proliferation in the presence of piericidin A.

Importantly, piericidin A and oligomycin were not used together since such association would generate a third component caused by the loss of membrane potential, which, in the presence of piericidin A alone, is maintained by the reverse of ATP synthase but would be inhibited by oligomycin. The investigation into the effect of the membrane potential on astrocytes’ growth and viability would make this study out of the current scope. The use of inhibitors downstream of complex I was also not evaluated due to the complexity of the approach. The inhibition of complexes III and IV affects the dihydroorotate dehydrogenase pathway involved in ubiquinone-mediated de novo pyrimidine synthesis [49,50,51]. Thus, compounds inhibiting mitochondrial respiration at sites downstream to complex I would impair cell viability and proliferation by a mechanism other than bioenergetics.

When astrocytes cultured in the absence or presence of mitochondrial inhibitors were evaluated concerning glucose consumption and lactate production (Figure 6), the mass of lactate release was similar that of glucose consumed, regardless of the condition. This may suggest that all the glucose consumed is converted into lactate. However, lactate can also be from other sources, such as glutamine [52,53]. Accordingly, glucose can follow other fates besides lactate [54,55]. Nevertheless, considering the demand for lactate at the lactate shuttle between astrocytes and neurons under physiological conditions, it remains reasonable to suppose that most glucose consumed by astrocytes is converted to lactate [1,5,54]. The inhibition of mitochondrial ADP phosphorylation or respiratory chain strongly stimulated glucose consumption and lactate release in cultured astrocytes. Accordingly, previous studies have shown that the inhibition of mitochondrial respiration increased the rate of glycolysis in astrocytes but not neurons [56]. 

Finally, ATP regeneration by OXPHOS and by glycolysis was calculated based on oligomycin-sensitive oxygen consumption (5 ATP per O_2_) (Figure 2), the amount of lactate released (one ATP per lactate) (Figure 6), and actual mean cell number. According to these measurements, 179.2 ± 57.4 pmol ATP/sec/10^6^ cells (N = 7) are generated by glycolysis in proliferating astrocytes cultivated in control conditions, and 220.2 ± 49.0 pmol ATP/sec/10^6^ cells (N = 4) are produced during oxidative phosphorylation at the same conditions. In this sense, glycolysis sustains approximately 45% of the cellular ATP yielding in proliferating astrocytes. 

This study has certain limitations. Our proliferating astrocytes in primary culture are a suitable model to address the question whether mitochondrial oxidative metabolism is required by reactive and proliferative astrocytes. However, this model is not representative of neuron-glial cultures, organotypic slices, or the whole brain. Even if GFAP-positive astrocytes are suggestive of a reactive phenotype, GFAP and morphology alone are not considered sufficient to qualify astrocytes’ reactivity [57]. Moreover, our proliferating astrocytes were from mice pups, and the metabolism of proliferating cells from newborns might be different from the metabolism of non-newborn cells.

## 5. Conclusions

Here, we demonstrated that astrocytes sustainably proliferate when their energy metabolism relies only on aerobic glycolysis since their growth and survival do not require electron flux through respiratory complex I or oxidative phosphorylation. The glycolytic profile of these astrocytes could sustain the cell proliferation in the absence of mitochondrial respiration. Previous studies have shown that mature astrocytes are naturally glycolytic cells that can survive by aerobic glycolysis for an extended period without functional impairments [18]. In this sense, the present study extends the concept previously established in mature astrocytes to proliferating ones.

## Data Availability

The data presented in this study are available in this article and are available from the corresponding author upon request.

## Supplementary Materials

The following supporting information can be downloaded at: https://www.mdpi.com/article/10.3390/cells12050683/s1, Figure S1: Maximum oxygen consumption rate (OCR) of astrocytes in suspension.
