# Supplementary material for: Proliferating Astrocytes in Primary Culture Do Not Depend upon Mitochondrial Respiratory Complex I Activity or Oxidative Phosphorylation
Source: Cells. 2023 Feb 21;12(5):683. doi: 10.3390/cells12050683 (PMC10001222; doi:10.3390/cells12050683)
Supplement: Supplementary file 1 [file cells-12-00683-s001.zip › cells-2146869-supplementary.pdf]

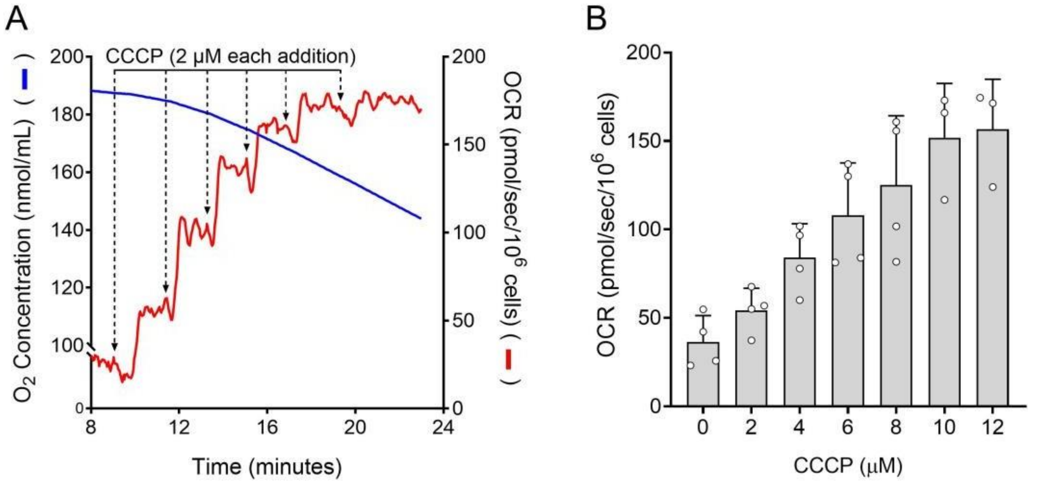

Figure S1. Maximum oxygen consumption rate (OCR) of astrocytes in suspension. Astrocytes (7.5 × 10^5/mL) were suspended in Medium 3 containing 20 mM HEPES-Na^+ and the OCR was monitored in a high-resolution oxygraph. **A**) Representative trace of OCR in suspended astrocytes. As indicated by the arrows, additions of 2 µM CCCP were made until reaching the OCR maximum. **B**) OCR values under different concentrations of CCCP (2–12 µM).
